# Supplementary figures and images for: Child marriage in rural Bangladesh and impact on obstetric complications and perinatal death: Findings from a health and demographic surveillance system
Source: PLoS One. 2023 Jul 19;18(7):e0288746. doi: 10.1371/journal.pone.0288746 (PMC10355438; doi:10.1371/journal.pone.0288746)

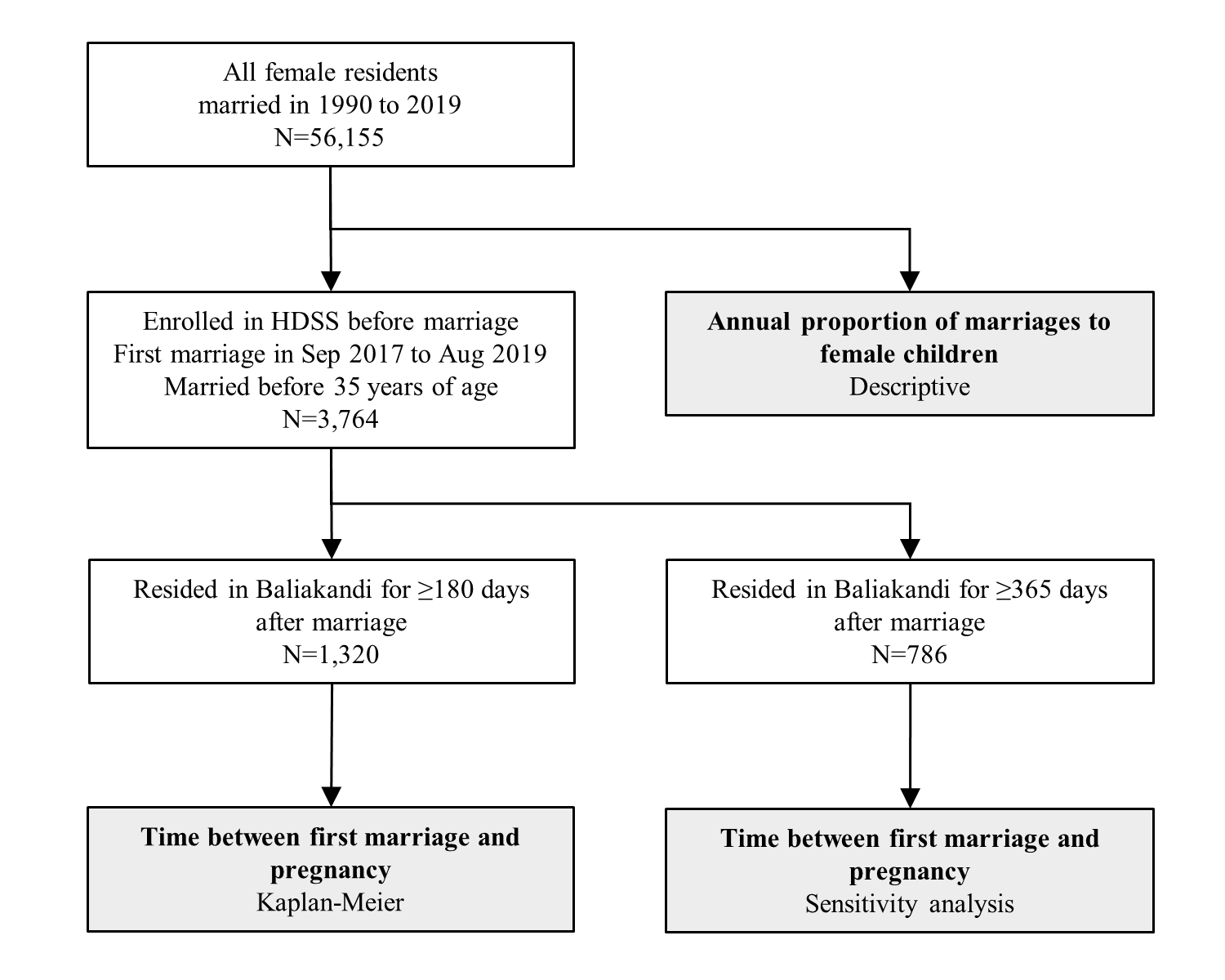

Supplement: S1 Fig — (TIF) [file pone.0288746.s001.tif]

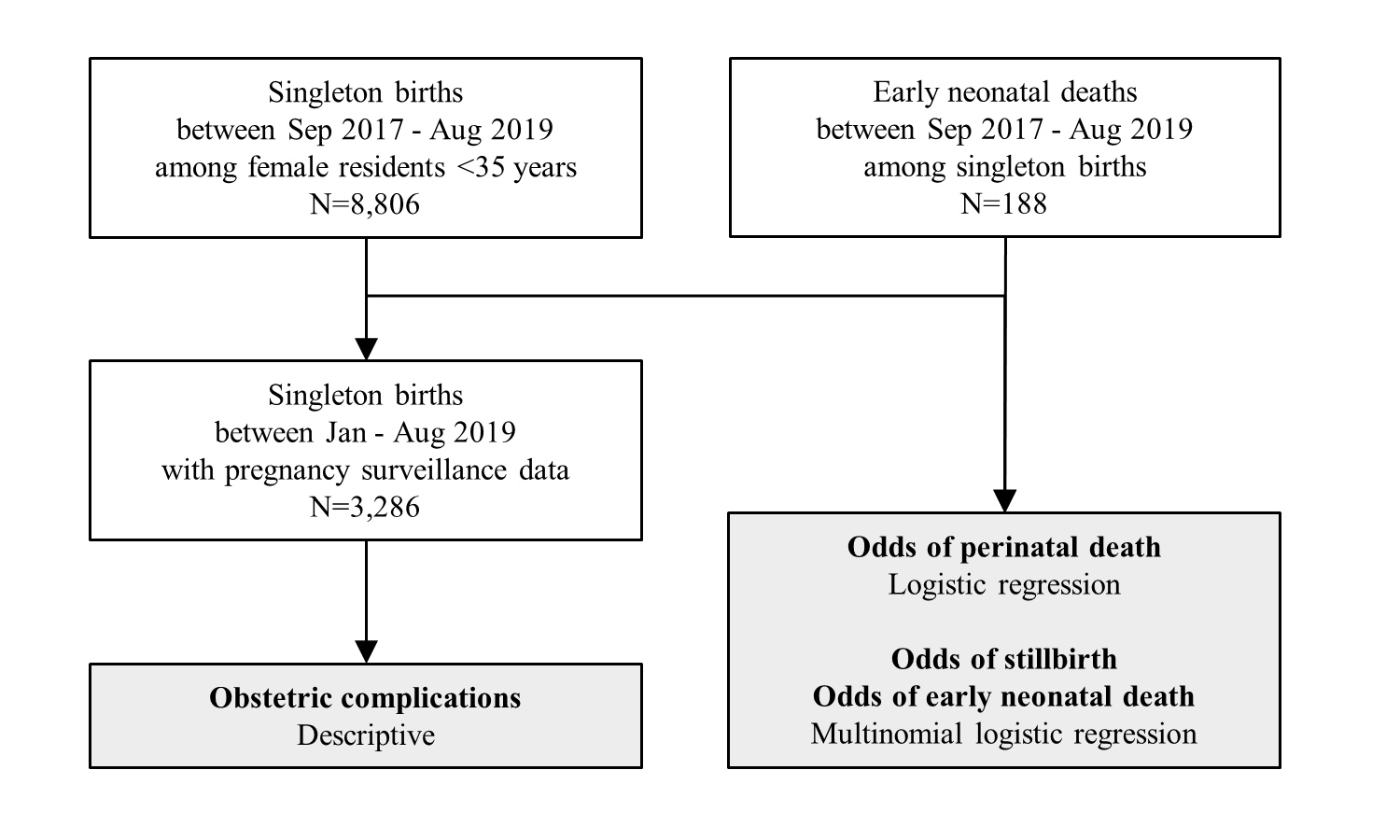

Supplement: S2 Fig — (TIF) [file pone.0288746.s002.tif]
