# Supplementary material for: Child marriage in rural Bangladesh and impact on obstetric complications and perinatal death: Findings from a health and demographic surveillance system
Source: PLoS One. 2023 Jul 19;18(7):e0288746. doi: 10.1371/journal.pone.0288746 (PMC10355438; doi:10.1371/journal.pone.0288746)
Supplement: S1 Table — (DOCX) [file pone.0288746.s003.docx]

# S1 Table. Excerpt from birth history questionnaire

| **Q #** | **Questions** | **Coding categories** | | | | | | | | **SKIP** |
| --- | --- | --- | --- | --- | --- | --- | --- | --- | --- | --- |
| 103 | When have you got married? Write year and month | Month | \|____\|____\| | | | | | | |  |
|  |  | Year | \|____\|____\|____\|____\| | | | | | | |  |
|  |  | Don’t know | 99 | | | | | | |  |
| 104 | Have you ever given birth? | Yes……………………………………………….…… | | | | | 1 | | |  |
|  |  | No……………………………………………….……. | | | | | 2 | | | 🡪109 |
| 105 | Do you have any sons or daughters to whom you have given birth and who are now living with you? | Yes………………………………………………….… | | | | | 1 | | |  |
|  |  | No…………………………………………………….. | | | | | 2 | | | 🡪107 |
| 106 | How many sons live with you?  And  How many daughters live with you? | Number of sons living with you | | \|___\|___\| | | | | | |  |
|  |  | Number of daughters living with you | | \|___\|___\| | | | | | |  |
| 107 | Do you have any sons or daughters to whom you have given birth who are still alive but do not live with you? | Yes……………………………………………………. | | | | | | 1 | |  |
|  |  | No…………………………………………………….. | | | | | | 2 | | 🡪109 |
| 108 | How many sons are alive but do not live with you?  And  How many daughters are alive but do not live with you? | Number of sons living elsewhere. | | \|___\|___\| | | | | | |  |
|  |  | Number of daughters living elsewhere .......... | | \|___\|___\| | | | | | |  |
| 109 | Have you ever given birth to a boy or a girl who was born alive but later died?  IF NO, PROBE: Any baby who cried or showed any sign of life but only survived a few hours or days? | Yes……………………………………………………. | | | | | 1 | | |  |
|  |  | No…………………………………………………….. | | | | | 2 | | | 🡪111 |
| 110 | In all, how many boys have died?  How many girls have died? | Number of boy child died | | | \|___\|___\| | | | | |  |
|  |  | Number of girl child died | | | \|___\|___\| | | | | |  |
| 111 | Some pregnancies end before full term as miscarriage or an abortion, while other may result in a stillbirth.  Have you had any pregnancies that did not result in live births? | Yes……………………………………………………. | | | | | 1 | | |  |
|  |  | No…………………………………………………….. | | | | | 2 | | | 🡪113 |
| 112 | In all, how many pregnancies did not result in a live birth? | Number……………………………………….. | | \|___\|___\| | | | | | |  |
| 113 | Sum answers 106, 108, 110, AND 112 and enter total | Number of total pregnancy outcome…………. | | \|___\|___\| | | | | | |  |
| 114 | CHECK 113 Just to make sure that I have this is correct: you have had  ______# children still alive (Q106+Q108)  ______# children have died (Q110), and  ______# pregnancies which did not result in a live birth (Q112)?  Is that correct? | Yes…………………………………………………….. | | | | | | | 1 | 🡪Stop |
|  |  | No……………………………………………………… | | | | | | | 2 |  |
|  |  | If NO ,probe and correct Q105 – 113 as necessary | | | |  | | | |  |
| 115 | Are you pregnant? | Yes………………………………………………….. | | | | 1 | | | |  |
|  |  | No…………………………………………………… | | | | 2 | | | | 🡪Stop |
|  |  | Don’t know…………………………………………. | | | | 9 | | | | 🡪Stop |
| 116 | What is the date when your last menstruation started? | \|__\|__\|-\|__\|__\|-\|__\|__\|__\|__\| | | | |  | | | |  |
