# Supplementary material for: Child marriage in rural Bangladesh and impact on obstetric complications and perinatal death: Findings from a health and demographic surveillance system
Source: PLoS One. 2023 Jul 19;18(7):e0288746. doi: 10.1371/journal.pone.0288746 (PMC10355438; doi:10.1371/journal.pone.0288746)
Supplement: S2 Table — (DOCX) [file pone.0288746.s004.docx]

# S2 Table. Excerpt from pregnancy surveillance questionnaire

| **Q #** | **Question** | **Coding Category** | | | |
| --- | --- | --- | --- | --- | --- |
| 4.1 | Did you have any of the following health problems during labor and delivery?  Obstructed labor/prolong labor/failure to progress delivery  Heavy bleeding during delivery  Heavy bleeding after delivery  High fever with severe abdominal pain  High fever with smelly discharge  High blood pressure  Severe headache with blurred vision  Convulsion  Fetal malpresentation (transverse, oblique, breech lie)  Injury in the birth passage  Urine or feces coming out from birth passage  Oedema feet/face/body  Retained placenta  (Read each problem) |  | Yes | No | Don’t know |
|  |  | 1. Obstructed labor/prolong labor/failure to progress delivery | 1 | 2 | 8 |
|  |  | 1. Heavy bleeding during delivery | 1 | 2 | 8 |
|  |  | 1. Heavy bleeding after delivery | 1 | 2 | 8 |
|  |  | 1. High fever with severe abdominal pain | 1 | 2 | 8 |
|  |  | 1. High fever with smelly discharge | 1 | 2 | 8 |
|  |  | 1. High blood pressure | 1 | 2 | 8 |
|  |  | 1. Severe headache with blurred vision | 1 | 2 | 8 |
|  |  | 1. Convulsion | 1 | 2 | 8 |
|  |  | 1. Fetal malpresentation (transverse, oblique, breech lie) | 1 | 2 | 8 |
|  |  | 1. Injury in the birth passage | 1 | 2 | 8 |
|  |  | 1. Urine or feces coming out from birth passage | 1 | 2 | 8 |
|  |  | 1. Oedema feet/face/body | 1 | 2 | 8 |
|  |  | 1. Retained placenta | 1 | 2 | 8 |
| 4.2 | Were you admitted to a hospital during delivery? | Yes …………………………………………………………………1  No ………………………………………………………………….2 SKIP🡪4.3 | | | |
| 4.2a | If yes, why were you admitted to the hospital? | Write the reasons for hospital admission: | | | |
| 4.2b | Did you plan earlier to get admitted to the hospital? | Yes …………………………………………………………………1  No …………………………………………….……………………2 | | | |
| 4.3 | Did you receive any of the following management during this delivery?  Blood transfusion  Hysterectomy  Episiotomy  (Read each condition) |  | Yes | No | Don’t know |
|  |  | 1. Blood transfusion | 1 | 2 | 8 |
|  |  | 1. Hysterectomy | 1 | 2 | 8 |
|  |  | 1. Episiotomy | 1 | 2 | 8 |
