# Supplementary material for: Child marriage in rural Bangladesh and impact on obstetric complications and perinatal death: Findings from a health and demographic surveillance system
Source: PLoS One. 2023 Jul 19;18(7):e0288746. doi: 10.1371/journal.pone.0288746 (PMC10355438; doi:10.1371/journal.pone.0288746)
Supplement: S3 Table — (DOCX) [file pone.0288746.s005.docx]

# S3 Table. Characteristics of female residents under 35 years of age who had first marriages, Baliakandi sub-district, Bangladesh, September 2017 to August 2019.

| **Characteristic** | **All**  **N=3,764**  **n (%)** | **Remained in Baliakandi ≥180 days after marriage**  **N=1,320**  **n (%)** |
| --- | --- | --- |
| Age at marriage (years) |  |  |
| 10 to 15 | 1077 (29%) | 433 (33%)^1^ |
| 16 to 17 | 1150 (31%) | 376 (28%) |
| 18 to 34 | 1537 (41%) | 511 (39%) |
| Household wealth (quintile) |  |  |
| Highest | 733 (19%) | 295 (22%) |
| High | 767 (20%) | 277 (21%) |
| Middle | 770 (20%) | 277 (21%) |
| Low | 759 (20%) | 259 (20%) |
| Lowest | 734 (20%) | 211 (16%) |
| Pregnant by August 2019^2^ | 786 (21%) | 715 (54%) |

^1^Only 12-15 years for subgroup

^2^Missing pregnancies from residents who out-migrated before end of August 2019
