# Supplementary material for: Child marriage in rural Bangladesh and impact on obstetric complications and perinatal death: Findings from a health and demographic surveillance system
Source: PLoS One. 2023 Jul 19;18(7):e0288746. doi: 10.1371/journal.pone.0288746 (PMC10355438; doi:10.1371/journal.pone.0288746)
Supplement: S4 Table — Adjusted model restricted to nulliparous mothers and adjusted for maternal age and household wealth. Total 8,806 singleton births among female residents under 35 years at birth, Baliakandi sub-district, Bangladesh, September 2017 to August 2019. (DOCX) [file pone.0288746.s006.docx]

# S4 Table. Crude and adjusted odds ratios of stillbirth and early neonatal death, compared to live births surviving more than 7 days. Adjusted model restricted to nulliparous mothers and adjusted for maternal age and household wealth. Total 8,806 singleton births among female residents under 35 years at birth, Baliakandi sub-district, Bangladesh, September 2017 to August 2019.

|  | **Stillbirth**  **Crude odds ratio**  **(95% CI)** | **Early neonatal death**  **Crude odds ratio**  **(95% CI)** | **Stillbirth**  **Nulliparous model**  **Adjusted odds ratio (95% CI)** | **Early neonatal death**  **Nulliparous model**  **Adjusted odds ratio (95% CI)** |
| --- | --- | --- | --- | --- |
| Maternal age at delivery (years) |  |  |  |  |
| 13 to 15 | 2.23 (1.19-4.16) | 1.28 (0.56-2.93) | 1.77 (0.92-3.41) | 0.96 (0.41-2.24) |
| 16 to 17 | 1.23 (0.77-1.98) | 1.57 (1.01-2.42) | 1.05 (0.63-1.74) | 1.10 (0.67-1.81) |
| 18 to 34 | Ref | Ref | Ref | Ref |
| Household wealth quintile |  |  |  |  |
| Highest | - | - | 0.51 (0.26-1.01) | 0.52 (0.27-0.97) |
| High | - | - | 0.85 (0.47-1.54) | 0.46 (0.24-0.88) |
| Middle | - | - | 0.71 (0.38-1.32) | 0.64 (0.35-1.16) |
| Low | - | - | 0.94 (0.51-1.72) | 0.74 (0.41-1.35) |
| Lowest | - | - | Ref | Ref |
