# Supplementary material for: Child marriage in rural Bangladesh and impact on obstetric complications and perinatal death: Findings from a health and demographic surveillance system
Source: PLoS One. 2023 Jul 19;18(7):e0288746. doi: 10.1371/journal.pone.0288746 (PMC10355438; doi:10.1371/journal.pone.0288746)
Supplement: Attachment — Submitted filename: Response to reviewers.docx [file pone.0288746.s009.docx]

We thank the reviewers for their feedback and comments. Please find our response to the comment listed by Reviewer #2.

**Reviewer #2: The authors have addressed all comments well. While I have no concerns and its not necessary to make any edits, the one thing that might be helpful would be to provide an assessment in the discussion of how generalizable these results from a fairly small rural sub-district might be to all of rural Bangladesh.**

We have added a statement on generalizability in the discussion section [lines 319-322].
